# Amputation at the Ankle Joint

**Published:** 1845-02

**Authors:** 


*Amputation at the Ankle Joint.*—This operation, Mr. Syme says, in the *London and Edinburgh Medical Journal*, should supersede amputation below the knee in a great many cases. The flap should be obtained from the heel and sole of the foot, as the natural thickness of the integument at those parts will afford a proper support to the stump. The best instrument for performing the operation is a large bistoury, or small amputating knife, with a blade about four inches long. There is no occasion for a tourniquet, as the assistant has complete command of the vessels by grasping the ankle. The incisions across the instep and sole of the foot should be curved, with the convexity forwards, and exactly opposite each other. A line drawn round the foot, midway between the head of the fifth metatarsal bone and the malleolus externus, will show their extent anteriorly, and they should meet a little way further back, opposite the malleolar projections of the tibia and fibula. If the ankle-joint is sound, the malleolar processes should be removed by cutting pliers; but if the articulating surfaces are deceased, a thin slice thereof should be sawn off. Care should be taken to avoid cutting the posterior tibial artery before it divides into the plantar branches, as in two cases where Mr. Syme did so, there was partial sloughing of the flap. The edges of the wound should be stitched together, and lightly dressed. When the cure is completed, the stump is of a conical form, the thick integument of the heel constituting its apex, or point of compression.

